# Inclusion of the US Preventive Services Task Force Recommendation for Mammography in State Comprehensive Cancer Control Plans in the US

**DOI:** 10.1001/jamanetworkopen.2022.9706

**Published:** 2022-05-02

**Authors:** Mehrnoosh Soori, Elizabeth A. Platz, Otis W. Brawley, Robert S. Lawrence, Norma F. Kanarek

**Affiliations:** 1 Department of Epidemiology, Johns Hopkins University Bloomberg School of Public Health, Baltimore, Maryland; 2 Department of Oncology, Johns Hopkins University School of Medicine, Sidney Kimmel Comprehensive Cancer Center at Johns Hopkins, Baltimore, Maryland; 3 Department of Environmental Health and Engineering, Johns Hopkins University Bloomberg School of Public Health, Baltimore, Maryland

## Abstract

**Importance:**

The recommendations for the age and frequency that women at average risk for breast cancer should undergo breast cancer mammography screening have been a matter of emotional, political, and scientific debate over the past decades. Multiple national organizations provide recommendations for breast cancer screening age and frequency. US Centers for Disease Control and Prevention (CDC) funding for state comprehensive cancer control (CCC) planning requires compliance with stated objectives for attaining goals. US Preventive Services Task Force (USPSTF) recommendations on cancer prevention and control are currently used to require coverage of prevention services.

**Objectives:**

To evaluate the consistency of state CCC plan objectives compared with the most current (2016) USPSTF recommendations for the age and frequency that individuals should undergo mammography screening and to make recommendations for improvement of state CCC plans.

**Design, Setting, and Participants:**

This cross-sectional study used a descriptive, point-in-time evaluation and was conducted from November 1, 2019, to June 30, 2021. In November 2019, the most recent CCC plans from 50 US states and the District of Columbia were downloaded from the CDC website. The recommended ages at which to begin and end mammography examinations and the frequency of mammography examinations were extracted from plan objectives.

**Main Outcomes and Measures:**

The recommendations found in CCC plan objectives regarding the ages at which to begin and end mammography examinations and the frequency of mammography examinations for women with average risk for breast cancer were compared with USPSTF recommendations.

**Results:**

Of the 51 CCC plans, 16 (31%) were consistent with all USPSTF recommendations for age and frequency that women at average risk should undergo mammography. Twenty-six plans (51%) were partially consistent with recommendations, and 9 plans (18%) were not consistent with any of the 3 guideline components.

**Conclusions and Relevance:**

Compared with the USPSTF recommendation, state CCC plans are not homogenous regarding the age and frequency that women at average risk for breast cancer should undergo mammography. This variation is partially due to differences in state-specific planning considerations and discretion, variations in recommendations among national organizations, and publication of plans prior to the most current USPSTF recommendation (2016). Specifying the concept that high-risk populations need different age and frequency of screening recommendations than the general population may reduce heterogeneity among plans.

## Key Points

**Question:**

Are the objectives of US state comprehensive cancer control (CCC) plans consistent with the current US Preventive Services Task Force (USPSTF) recommendation for mammography for women at average risk for breast cancer?

**Findings:**

This cross-sectional study found that the objectives of CCC plans from 50 US states and the District of Columbia do not universally suggest that USPSTF-recommended screening should start at the age of 50 years for women at average risk for breast cancer. Many state CCC plan objectives do not include an age for ending screening, and a few do not include frequency of screening.

**Meaning:**

This study suggests that specifying that women at high risk require different screening age recommendations may reduce the heterogeneity in mammography guidance that is present in state CCC plans.

## Teaser

This cross-sectional study evaluates the consistency of state comprehensive cancer control plan objectives compared with the 2016 US Preventive Services Task Force (USPSTF) recommendations for the age and frequency that women should undergo mammography.

## Introduction

In the US, comprehensive cancer control (CCC) plans are unique documents that include overall goals, measurable objectives, and evidence-based strategies to address the most common and most intervenable types of cancer in in each US state. These state CCC plans are often developed with multisector stakeholders for 4- to 6-year periods and should be updated on a regular basis.^1^ Centers for Disease Control and Prevention (CDC) funding requires the inclusion of state cancer screening prevalence rates and specific objectives and strategies for cancer screening in CCC plans,^2^ in addition to other statistics that are aimed at improving cancer outcomes and motivating effort.

Breast cancer is the most common cancer and the second-highest cause of cancer death among women in the US.^3^ Breast cancer can be detected by mammography at early stages, when fatality is lowest. A program of routine, high-quality screening has been shown to reduce mortality by 25% to 31% for women aged 50 to 69 years,^4^ and thus it is an effective intervention for this age-targeted group. Several organizations have evaluated the evidence and have sought consensus in guidelines for the use of mammography by women at average risk for breast cancer; these organizations include the American Cancer Society (2021; annual screening at 45-55 years of age and biennial screening at 55 years or older),^5^ the US Preventive Services Task Force (USPSTF) (2016; biennial screening at 50-75 years of age),^6^ and at least 2 medical associations, the American College of Physicians (2019; biennial screening starting at 50 years until 10 years of life expectancy)^7^ and the American Academy of Family Physicians (2016; biennial screening at 50-75 years of age).^8^ The US Patient Protection and Affordable Care Act of 2010 stipulates required coverage of prevention services that are graded by the USPSTF as an “A” or “B” recommendation.^6,9^ Other federal legislation further ensures that there is no copay associated with mammography for screening among women aged 40 to 49 years.^10^

All of the organizations with recommendations agree on the benefits of breast cancer screening for women at average risk. However, the ages at which women should start and end mammography examinations and the frequency of mammography examinations have been a matter of political, emotional, and scientific debate for 3 decades.^11^ In particular, the recommended age at which to start screening has changed over the years based on accumulated scientific evidence.^4,8^ Apart from the USPSTF, differences in recommendations arise from the different studies used for evidence to support the recommendations and the organizational criteria for weighing evidence, membership advocacy, and methods of harm as well as the benefit to harm ratio threshold calculations.^12^ Some health system concerns are overdiagnosis, false-positive results, and radiation harm caused by the increased number of mammography examinations for women who receive a diagnosis at a younger age. The calculation of benefits and harms varies based on age group and evidence for individual preferences and values, leading to more emphasis on shared decision-making between patients and clinicians,^6,13^ especially for those aged 40 to 49 years. The CDC expects state CCC plans to stay up to date with current recommendations,^2^ although, in practice, the periodicity of plan updates may preclude inclusion of the most current recommendations.^14^

The most current USPSTF recommendation statement (grade B) issued in 2016 states that women aged 50 to 74 years with average risk (no signs, symptoms, or prior diagnosis of breast cancer; no history of a high-risk breast lesion; no history of chest irradiation at a young age; and no predisposing genetic or family history profile) for developing breast cancer should undergo biennial mammography.^6^ Prior to 50 years of age, the decision to start mammography is an individual one. The USPSTF recommendation statements prior to 2009 recommended beginning biennial mammography at 40 years of age.^15^ The objective of this study is to evaluate the inclusion of the B-rated, USPSTF-recommended frequency of mammography examinations and the ages at which women at average risk should start and end mammography examinations in state CCC plans.

## Methods

This cross-sectional study used a descriptive, point-in-time evaluation and was conducted from November 1, 2019, to June 30, 2021. The CCC plans from 50 US states and the District of Columbia were downloaded from the CDC website^16^ in November 2019. The recommended frequency of mammography examinations and the recommended ages at which to begin and end mammography examinations in relevant CCC plan objectives were extracted. The recommended ages at which to start and end mammography examinations that are contained in the CCC plans were compared with the most current USPSTF (2016) recommendation.^6^ The number of states that recommended each screening age category was quantified in Excel (Microsoft Corp); the District of Columbia also had recommendations. The numbers and percentages of all of the US states and the District of Columbia are presented. Institutional review board review was not sought because this was not human participants research, as the observations were conducted among state CCC plans. This study followed the Strengthening the Reporting of Observational Studies in Epidemiology (STROBE) reporting guideline.

### Statistical Analysis

The numbers and percentages of the plans were derived using Excel, version 2018 (Microsoft 365 Apps for Enterprise). No statistical tests were performed.

## Results

The Table aggregates CCC plans based on the recommended ages at which women should start and end mammography examinations and the frequency of mammography examinations. Of 51 plans, 16 (31%) referenced the complete recommendation of conducting biennial mammography for women 50 to 74 years of age, meeting the recommendations for the ages at which women should start and end mammography examinations and the frequency of screening examinations. Just over half of all plans (26 of 51 [51%]) were partially consistent with recommendations, while 18% of the plans (9 of 51) were not consistent with any part of the USPSTF recommendation.

**Table.** Frequency of State Comprehensive Cancer Control Plans Meeting US Preventive Services Task Force Recommendation for Breast Cancer Screening (Aged 50-74 Years, Biennially), 50 US States and the District of Columbia

Outcome	Plans, No./total No. (%)
Met recommendation	16/51 (31)
Partially met recommendation	26/51 (51)
Starting at age of 40 y for screening, biennial frequency	15/26 (58)
Starting at the ages of both 40 and 50 y, biennial frequency^a^	4/26 (15)
No age specified for beginning screening, biennial frequency	1/26 (4)
No age specified for ending screening, biennial frequency	22/26 (85)
Screening at ages 50-75 y, frequency not specified	4/26 (15)
Met no part of the recommendation	9/51 (18)

^a^ One state included ages of 40, 50, and 52 years.

Among the plans that were partially consistent with USPSTF recommendations, 73% (19 of 26) recommended starting mammography at 40 years of age, and 31% (8 of 26) recommended starting mammography at 50 years of age. Most plans (85% [22 of 26]), did not specify the age at which women should stop getting mammography examinations. Fifteen percent of plans (4 of 26) did not specify the frequency of mammography examinations, and 15% (4 of 26) used another age at which women should start getting mammography examinations (3 plans specified 40 and 50 years, and 1 plan specified 40, 50, and 52 years). Among those plans that were only partially consistent with the USPSTF recommendation, the biennial frequency of mammography examinations was included in the objectives of 22 plans (85%).

Fifty-nine percent of plans (30 of 51) used the recommended starting age of 50 years, 37% of plans (19 of 51) used a starting age of 40 years (as aligned with the 2009 USPSTF recommendation), 8% of plans (4 of 51) included starting mammography examinations at both 40 and 50 years of age, and 1 plan (2%) included an additional starting age. In all, 20% of plans (10 of 51) did not specify an age at which women should begin undergoing mammography.

## Discussion

This analysis shows that incorporation of the current USPSTF recommendation into state CCC plans is incomplete regarding the the ages at which women should start and end mammography examinations and the frequency of mammography examinations. For a recommendation tied to service coverage, this is a serious gap in public health policy. It may also indicate a lack of consensus among agencies, such as state health departments and their partners in cancer prevention and control. There are several potential reasons for the lack of consensus.

First, CCC plans are written by and for the state, with the primary audience being state policy makers, individual residents, and organizations operating in the state. Comprehensive cancer control plans are expected to reflect the state’s unique burden of cancer. We evaluated the expectation that state plans follow the CDC’s advice to include a complete breast cancer screening objective using the current USPSTF recommendation, which is also tied to insurance coverage without cost sharing. Raising awareness among states of the importance of evidence-based guidelines may be needed.^14^

Second, the frequency of plan updates was periodic but still not often enough to always be relatively up to date on the most recently issued recommendations. Twenty percent (n = 10) of CCC plans were at least 5 years or more out of date at the time of abstraction relative to the 2016 USPSTF recommendation. State attention to CCC plan updates is imperative to maintain currency with emerging recommendations, although plan updates may not be enough. One solution is that plans point to the USPSTF recommendation with a hyperlink and state that, at any point in time, the current USPSTF recommendation may be updated.^6^

Third, controversies about breast cancer screening recommendations arising from debates dating back to the early 1980s have not abated,^11,17^ despite the Patient Protection and Affordable Care Act’s use of the USPSTF recommendations. The different rules for Medicare^10^ and Medicaid^18^ may also contribute to our inability to settle on a consensus recommendation across payers. Several cancer organizations also promote different recommendations.^5,6,8,19^ This lack of 1 message nationally may be replayed at the state level with advocates, program managers, policy makers, and scientists being at odds regarding use of 1 national, albeit evidence-based, recommendation put forth by the USPSTF. We would argue for the use of the USPSTF recommendation because it is tied to payment and thus improves screening access.

Fourth, the lack of completeness in mammography recommendations in state plans means that neither the general population nor any high-risk subpopulation is benefitting from the current knowledge base for age and frequency of appropriate screening. The current USPSTF recommendation defines “high-risk” status as women with a family history of breast cancer or *BRCA1/2* (OMIM 113705 and 600185) gene variants or chest radiation at younger than 30 years of age.^6^ Thus far, however, there is no 1 tool^12^ to determine high-risk status that may have a different benefit to harm ratio and that may lead to a more frequent number of examinations and narrower age range. Given the presumption that early age at screening is of a greater benefit than later age at screening for women at high risk, the decision to screen thus becomes an informed clinical decision (after discussion) for women 40 to 49 years of age who possess certain characteristics, such as *BRCA1/2* gene variants, family history of breast cancer, or chest radiation at younger than 30 years of age.^20^ Specifying the concept that a high-risk population needs a different age and frequency of screening recommendations may reduce heterogeneity in plans. A substantial proportion of breast cancer mortality is due to the quality and timing of treatment and obesity, in addition to screening. Maximal reduction in breast cancer mortality can be achieved by focusing on optimized treatment followed by high-quality, timely mammography and obesity prevention.^21^

### Limitations

This study has some limitations. This study was conducted at 1 point in time, although the timeline for writing state CCC plans occurred with state-determined and varying periodicity. Under the evaluation design, we did not contact CCC plan authors to understand the decision-making process regarding the objectives for breast cancer screening, and we did not account for CDC CCC plan guidance evolving over time. Every state in the US produced a plan in a unique context. Because coverage of prevention services without cost sharing is based on the USPSTF recommendation with grades A or B, we evaluated CCC plans against the USPSTF recommendation for breast cancer screening for average-risk populations. We recognize that implementation of population-based screening should be state and health care system specific.

## Conclusions

Despite great public interest in detectingbreast cancer early, when it may be more effectively treated, state CCC plans are not uniformly providing complete USPSTF recommendations for mammography examinations for women at average risk. State plans may be improved by (1) emphasizing a single, consistent public health message for the general population (women at average risk, 50-74 years of age, and biennial screening) and (2) explicitly distinguishing women at average risk from various high-risk populations of women who may be screened at younger ages after discussion with their physician. The former will require national organization consensus building. The latter will enable more appropriate, evidence-based use of mammography and other breast cancer prevention tools.^13,22^
